# Myxedema Psychosis: Diagnostic Challenges and Management Strategies in Hypothyroidism-Induced Psychosis

**DOI:** 10.7759/cureus.57259

**Published:** 2024-03-30

**Authors:** Moujib Omri, Mohamed Ferhi, Nina Lentz, Marizia Oliveira Galvao, Oliver Hamm

**Affiliations:** 1 Psychiatry and Psychotherapy Department, Klinikum Mutterhaus der Borromäerinnen, Trier, DEU; 2 Psychiatry Department, Ibn El Jazzar University Hospital, Kairouan, TUN

**Keywords:** psychiatric co-morbidity, antipsychotic medication, first episode psychosis, myxedema madness, thyroxine replacement therapy, hypothyroidism, hypothyroidism-induced psychosis, myxedema psychosis

## Abstract

Myxedema psychosis (MP), a rare psychiatric manifestation of hypothyroidism, presents significant diagnostic and therapeutic challenges. This case report details the presentation, diagnosis, and successful management of a 60-year-old woman with MP, who was initially admitted to the psychiatric department for new-onset psychosis following the cessation of hormone replacement therapy after a subtotal thyroidectomy performed 20 years prior. Despite the rarity of psychosis as an initial presentation of hypothyroidism, this case underscores the critical importance of considering endocrine disorders in the differential diagnosis of unexplained psychotic symptoms. The clinical findings included a polymorphic delusional system and auditory hallucinations, without significant abnormalities on magnetic resonance imaging. Elevated thyroid-stimulating hormone (TSH) levels confirmed hypothyroidism, leading to the diagnosis of MP. Treatment with l-thyroxine resulted in complete resolution of symptoms in three weeks, highlighting the efficacy of hormone replacement therapy. This case contributes to the limited literature on MP and echoes the need for awareness among clinicians to ensure timely and accurate diagnosis and treatment.

## Introduction

Hypothyroidism, a prevalent endocrine disorder that affects approximately 3.6% of the population, manifests itself through a spectrum of clinical symptoms, including notable neuropsychiatric features such as depression, anxiety, mania, and cognitive dysfunction [1]. Despite its commonality, the appearance of psychosis as an initial presentation of hypothyroidism remains a rarity in the medical literature, with few documented cases where the onset of hypothyroidism in a patient’s clinical picture is marked by psychotic symptoms [2,3]. This phenomenon, often referred to as ‘myxedema madness’, traces its nomenclature to a publication by Asher in 1949, which detailed 14 case reports of individuals who exhibited psychotic manifestations in conjunction with myxedema [4]. The intersection of hypothyroidism and psychosis dates even further, with the first descriptions emerging in 1888 by the Committee of the Clinical Society of London, which recognized psychosis as a possible complication of hypothyroidism [5]. Despite the more than seven decades since Asher’s descriptive analysis, and significant advances in our understanding and management of hypothyroidism, the knowledge surrounding myxedema psychosis (MP) remains limited [6]. The diagnosis of MP presents a significant challenge, as its symptoms can closely mimic those of primary psychiatric conditions, leading to potential oversight of an underlying hypothyroid etiology. This case report underscores the importance of considering hypothyroidism in the differential diagnosis of unexplained psychosis, highlighting the need for a vigilant and comprehensive diagnostic approach in such cases.

## Case presentation

Patient information

We present a case involving a 60-year-old woman who has been admitted to the psychiatry and psychotherapy department in Klinikum Mutterhaus der Borromäerinnen, Trier, Germany, following a notable episode characterized by erratic behavior. The patient, a divorced mother living with her daughter, has an average socioeconomic background. In particular, her medical history is free of psychiatric conditions but includes a diagnosis of iatrogenic hypothyroidism, which developed as a consequence of a subtotal thyroidectomy performed 20 years prior, aimed at treating a thyroid nodule accompanied by goiter. The patient had been on hormone replacement therapy after surgery, which she withdrew a few months before the onset of psychiatric symptoms.

Clinical findings

The onset of psychiatric symptoms began a few months after stopping hormone replacement therapy, culminating in a psychotic syndrome characterized by a polymorphic delusional system that persisted for more than a month. The patient's delusions were varied, encompassing themes of persecution, poisoning (believing she could only consume food shared with another from the same dish), influence, enchantment, and the emission of a "positive universal healing energy." Hallucinatory experiences, predominantly audio-acoustic in nature, featured voices distinct in timbre and clarity, which were both commentative and imperative, addressing the patient in the second person. Additional delusions included thought theft and bodily and sexual transformation, indicating a poorly systematized set of delusions. This means they were unorganized and lacked logical connections. The individual demonstrated full adherence to the delusional content, albeit without emotional engagement.

Diagnostic assessment

On physical examination, the patient did not show remarkable findings. Magnetic resonance imaging (MRI) did not reveal significant anomalies, with the exception of a suggestive image of a pituitary colloid cyst. Laboratory tests indicated a markedly elevated thyroid-stimulating hormone (TSH) level at 69 mIU/L, while levels of thyroid hormones - free triiodothyronine (FT3) and free thyroxine (FT4) - were at the lower limit of the normal range. Complete blood count (CBC), comprehensive metabolic panel (CMP), lipid profile, liver function tests, electrolyte panel, and inflammatory markers were within normal limits.

The clinical presentation posed a diagnostic challenge; however, the onset of psychosis after the discontinuation of hormone replacement therapy, coupled with elevated TSH levels, strongly indicated hypothyroid-related psychosis as the most probable diagnosis. The absence of significant changes on MRI further supported this diagnosis. A differential diagnosis was a primary psychiatric disease that could be determined only by follow-up in case of relapse after discontinuing antipsychotics in the presence of normal thyroid hormones. Furthermore, Hashimoto's encephalopathy (HE) was considered less likely given its typical association with euthyroid status in patients with neuropsychiatric characteristics in the context of autoimmune hypothyroidism [7,8].

Therapeutic intervention and follow-up

The initial treatment consisted of lorazepam and olanzapine. However, in light of the patient's non-improvement and subsequent diagnosis of hypothyroidism, hormone replacement therapy with l-thyroxine was initiated and neuroleptic treatment was phased out. The patient demonstrated a favorable response to the reintroduction of hormone replacement therapy, with a complete resolution of the psychotic symptoms within three weeks. Follow-up care in an outpatient setting was marked by a return to the patient's premorbid state and normalization of thyroid function tests.

## Discussion

In this case report, we delineate the rare presentation of psychosis as an initial manifestation of hypothyroidism in a 60-year-old woman, underscoring the clinical entity historically termed 'myxedema madness'. This unique case adds to the sparse literature that illustrates the development of MP in the absence of a prior psychiatric history. Our findings echo the observations first documented by Asher in 1949 [4], but they also highlight the persistent diagnostic challenges that clinicians face when confronted with psychiatric symptoms potentially rooted in endocrine disorders.

The pathophysiology underlying MP remains enigmatic, with contemporary research suggesting a multifaceted interplay of neurometabolic activities as potential contributors. Hypothyroidism has been associated with an imbalance of tyrosine hydroxylase in the anterior locus coeruleus [9] and a notable concentration of triiodothyronine (T3) receptors within the amygdala and hippocampus [10]. These regions of the brain are essential for emotional regulation and behavioral integration. Furthermore, altered serotonin-mediated neurotransmission has been implicated, in conjunction with attenuation of cerebral regional blood flow and glucose metabolism [11,12]. 

HE, also known as steroid-responsive encephalopathy associated with autoimmune thyroiditis, emerges as a pivotal differential diagnosis in patients with hypothyroidism presenting with psychosis [13]. Unlike MP, where neurochemical alterations in the brain due to thyroid hormone deficiency predominate, neuropsychiatric manifestations in HE are attributed to an autoimmune response, independent of hypothyroid status [13]. This explains the excellent response to steroids in most cases of HE. Systematic reviews have revealed that a minority of HE patients exhibit clinical hypothyroidism, with the majority being euthyroid, further differentiating it from MP [7,8]. In the context of our patient, the rapid improvement in symptoms with l-thyroxine without the administration of steroids, coupled with the lack of significant MRI findings and the history of iatrogenic hypothyroidism, makes HE a less probable diagnosis. Nonetheless, the possibility of HE cannot be completely dismissed and warrants consideration should there be a recurrence of psychotic symptoms, particularly in the setting of normalized thyroid hormone levels.

A recent systematic review [14] revealed the multifaceted etiology of hypothyroidism. In particular, among cases with identified causes, primary hypothyroidism emerged as the most common diagnosis, representing 48% of the total. Within this category, Hashimoto's thyroiditis was the most common specific cause, accounting for 17.3% of cases, followed by a single instance of postpartum hypothyroidism, which made up 1.9%. Furthermore, surgical procedures, including total or partial thyroidectomy, were involved in 9.6% of hypothyroidism cases, a finding that aligns with the etiology observed in our current case.

Treatment of MP involves a nuanced approach, focusing on the management of hormonal and psychiatric symptoms. A recent systematic review of MP cases [15] highlighted that thyroxine administration can be conducted effectively both intravenously (IV) and orally. Although an exploratory comparison of the data did not definitively favor one method over the other, there was an indication that IV administration could lead to faster recovery. However, this potential benefit must be balanced with the increased risk of arrhythmias associated with IV thyroxine [16]. Interestingly, the use of steroids, common in the management of myxedema coma and HE, appears less crucial in MP. To manage acute psychosis symptoms of MP, clinicians often use short-term antipsychotic treatments at the start of the disease [15,17]. They are usually stopped during follow-up if symptoms do not recur. Importantly, beginning thyroxine replacement therapy can sometimes worsen psychotic symptoms in the first week [17]. To counteract this, antipsychotics and anxiolytics may be temporarily used together. Other treatment modalities have also been used in certain cases [18,19], including electroconvulsive therapy and antidepressant drugs such as trimipramine and escitalopram. This diversity in treatment approaches highlights the importance of a comprehensive and tailored treatment plan that considers both the hormonal and psychiatric aspects of MP.

As a single case study, the findings and observations reported are subject to the unique circumstances and clinical decisions that affect this case. This specificity restricts the generalizability of the conclusions drawn to the broader population. Another limitation is that we did not assess the severity of psychotic symptoms using a standardized scale, such as the Positive and Negative Syndrome Scale (PANSS). Furthermore, while the case provides valuable information on the treatment and management of MP using thyroxine and antipsychotic medications, these findings are based on cumulative case reports and lack the rigor of controlled clinical trials, which emphasizes the need for prospective research.

## Conclusions

Although MP represents a rare etiology, its inclusion in the differential diagnosis of new-onset psychosis is crucial due to its notably positive prognosis. The present case illustrates that hypothyroidism can be immediately identified by determining blood TSH levels and is effectively managed in a majority of patients through appropriate thyroid hormone replacement therapy. This underscores the importance of considering thyroid function tests in patients with unexplained psychotic symptoms, facilitating the early diagnosis and treatment of this reversible cause of psychosis.
